# Effectiveness of interventions to increase device-measured physical activity in pregnant women: systematic review and meta-analysis of randomised controlled trials

**DOI:** 10.1186/s12966-022-01379-w

**Published:** 2022-12-01

**Authors:** Kayleigh J. Sharp, Lauren B. Sherar, Victoria E. Kettle, James P. Sanders, Amanda J. Daley

**Affiliations:** Centre for Lifestyle Medicine and Behaviour (CLiMB), School of Sport Exercise and Health Sciences, Loughborough University, Leicestershire, LE11 3TU UK

**Keywords:** accelerometers, device measured, gestational weight gain, pedometers, steps

## Abstract

**Background:**

Interventions that provide pregnant women with opportunities to access and participate in physical activity have been shown to be beneficial to their health. Much of this evidence however has been based on self-reported physical activity data, which may be prone to inflated effects due to recall bias and social desirability bias. No previous synthesis of randomised controlled trials has assessed the effectiveness of these interventions using only device measured data, to assess their health benefits more accurately in pregnant women. This systematic review and meta-analysis aimed to address this evidence gap.

**Data sources:**

Cochrane Central Register of Controlled Trials, Medline, SportDiscus, APA PsycINFO, Embase and Web of Science databases were queried from inception up to December 2, 2021. An updated search of PubMed was conducted on May 16, 2022.

**Study eligibility criteria:**

Randomised controlled trials that recruited pregnant women, participating in any physical activity intervention (excluding interventions aimed entirely at body conditioning), compared with standard antenatal care (comparators), using device-measured total physical activity as an outcome were eligible for inclusion.

**Methods:**

3144 titles and abstracts were screened for eligibility, and 18 met the inclusion criteria. Data were analysed using random effect models, (standardised mean difference and mean difference), using data from baseline to last available follow-up (primary end point), and until between 24 to 30 weeks gestation. Gestational weight gain was also assessed at these timepoints in the included trials.

**Results:**

No significant differences between the groups were found for total physical activity at last available follow-up or 24 to 30 weeks gestation (95% CI 0.03 to 0.27, *p* = 0.10: 95% CI -0.05 to 0.33, *p* = 0.15) respectively. On average, pregnant women randomised to a physical activity intervention completed 435 and 449 more steps per day than comparators at last available follow-up and at 24 to 30 weeks gestation (95% CI -0.5-870.6, *p* = 0.05: 95% CI 5.5-892.7, *p* = 0.05) respectively. Intervention participants also gained 0.69 kg less (95% CI -1.30 to -0.08, *p* = 0.03) weight than comparators.

**Conclusion:**

Based on device-measured data, interventions to promote physical activity during pregnancy have small but important effects on increasing physical activity and managing excessive gestational weight gain.

**Supplementary Information:**

The online version contains supplementary material available at 10.1186/s12966-022-01379-w.

## Background

According to the World Health Organisation (WHO) [1], women are advised to achieve at least 150 minutes of moderate-intensity physical activity per week during pregnancy. Adherence to these guidelines is associated with a range of physical and psychological health benefits, such as reduced risk of excessive gestational weight gain, improved sleep, increased likelihood of delivering an infant sized appropriately for gestational age, reduced obstetric comorbidities, and reduced anxiety and depressive symptoms [2–4]. Despite these benefits, evidence shows that only 32% of pregnant women achieve the recommended guidelines for physical activity in early pregnancy with this figure reducing to 12% in late pregnancy [5].

For cost reasons, ease of access, and expansive reach of participants, physical activity trials during pregnancy have typically measured physical activity via self-report questionnaires, such as the pregnancy physical activity questionnaire (PPAQ) [6]. For example, a recent systematic review of 15 randomised controlled trials (RCTs) that aimed to promote physical activity during pregnancy found these interventions to have only a small impact on reported activity levels [7], but 80% of the included studies collected data using self-report. Considering the limitations associated with self-report measures, such as inaccurate recall and self-report bias, this finding may not accurately reflect the physical activity levels of pregnant women. Recent technological advancements and the availability of wearable physical activity tracking devices (e.g., accelerometers and pedometers) have provided researchers with the opportunity to collect more objective data to assess the effectiveness of promoting physical activity, reducing the concerns about recall dependency and data accuracy of self-report measures [8]. It is important to have robust evidence on this question in pregnant women, especially given WHO [9] has a target to disseminate physical activity recommendations and promote the accessibility of appropriate support for physical activity for pregnant women by 2030.

### Objectives

To date, no evidence synthesis has focused exclusively on investigating the effectiveness of interventions to increase device-measured physical activity in pregnant women. This systematic review and meta-analysis aimed to address this literature gap and comprehensively synthesise evidence from RCTs about the effectiveness of interventions to increase participation in physical activity (device-measured) and manage gestational weight gain in pregnant women.

## Methods

This systematic review and meta-analysis was conducted according to PRISMA guidelines [10] (see Table S1) and the protocol was registered with the international prospective register of systematic reviews (PROSPERO) on 7 February 2022 (CRD42022308657).

### Eligibility criteria

Studies were eligible for inclusion if they were RCTs or quasi-RCTs [hereafter referred to as RCTs] that recruited pregnant women aged ≥18 years, including those with or without high-risk pregnancy (i.e., multiple births, existing health conditions, living with overweight or obesity). Any physical activity intervention (frequency, intensity, time, and type), in any setting was eligible for inclusion, although interventions exclusively aimed at body conditioning (e.g., yoga and tai-chi) were excluded because these types of interventions focus on posture, breath and meditation [11]. Trials were required to report data related to participation in the outcome of total physical activity (average of all intensities of physical activity combined) from baseline to final follow-up or provide data that allowed this outcome to be calculated. For this reason, trials reporting only participation in moderate-to-vigorous physical activity (MVPA), without data on participation in light physical activity were excluded. Only trials that had used a device to measure physical activity as an outcome in both trial groups were eligible. A full description of the eligibility criteria is available in Table S2.

### Information and search strategy

A systematic search of Cochrane Central Register of Controlled Trials (CENTRAL), Medline (EBSCO), SportDiscus (EBSCO), APA PsycINFO (EBSCO), Embase and Web of Science was conducted on December 2, 2021. Additional searches were also conducted in BASE (Bielefeld Academic Search Engine) on December 2, 2021. A 6-month search of PubMed was conducted on May 16, 2022, to ensure no recently published trials were missed [12]. The reference lists of relevant studies and previous reviews were searched to identify other potentially relevant studies. The search strategy was designed and tested in Medline (EBSCO) and adapted for the remaining databases (Table S3). No population, language, or age restrictions were applied, and no date limitations were included. Studies returned by the search strategy were imported and stored in the Covidence [13] (Veritas Health Innovation, Melbourne, Australia) online program.

### Trial selection and data collection process

Duplicates were identified through Covidence and automatically removed. The title and abstracts of the remaining RCTs were screened for eligibility using Covidence by three independent review authors with each trial screened by two of the three review authors (KJS, AJD, VEK). Conflicts were resolved through discussion and consensus, and if conflict persisted, a third independent reviewer was consulted. The full texts were screened independently by three of the review authors using the inclusion and exclusion criteria (Table S2) with every trial screened by two of the three review authors (KJS, AJD, LBS) and conflicts resolved as above. For studies not written in English, Google Translate was used; however, where Google Translate was unable to translate the document, an outsourced individual was contacted. Corresponding authors were contacted by email if further information was required.

### Trial details

The following information was extracted from each trial: general study information, participant information, intervention, comparators, outcomes included in this review and measurement, as detailed in Table S4 [14–31]. One reviewer extracted the information for the trial characteristics table (KJS). Three review authors independently extracted the outcome data with disagreements resolved through discussion and consensus (KJS, VEK, JPS).

### Risk of bias in individual trials

Two independent reviewers (from KJS, VEK, JPS) assessed the risk of bias (RoB) using the Cochrane RoB Tool v2 [32] which uses, a domain-based evaluation in five key areas: randomisation (D1), deviation from the intended interventions (D2), missing outcome data (D3), measurement of outcome (D4), and selective outcome reporting (D5). For incomplete data, a high RoB was defined as an attrition of ≥25%. Studies not reporting data cut-offs for valid device wear time were identified as having a high RoB. Disagreements between reviewers were discussed and resolved through consensus by referring to the full text. Where disagreements persisted, a third independent reviewer was consulted.

### Outcomes

The primary outcome was standardised mean difference (SMD) of the change in total physical activity (all intensities combined) between the groups at baseline and last available follow-up. Where studies included both steps per day and another assessment of total physical activity (e.g., Bisson et al. [16] reported steps per day and accelerometer counts per day), steps per day was prioritised for inclusion in the meta-analysis for the primary outcome of total physical activity. The secondary outcomes were mean difference (MD) in the change in minutes of MVPA and MD in the change in body weight (kg) from baseline to last available follow-up. The analyses of change in minutes of MVPA were only analysed in trials that had also provided data for total physical activity. Additionally, after post-registration consideration, change in total physical activity (SMD), steps per day (MD) and MVPA (MD) were explored between the groups, specifically between baseline and 24 to 30 weeks gestation. This is because previous research has suggested that pregnant women reduce their physical activity levels near the end of pregnancy; therefore, it was considered that physical activity participation during mid-pregnancy could produce different results. Furthermore, post-registration subgroup analyses were conducted to compare multi-component lifestyle interventions to those that were exclusively physical activity based for total physical activity and between interventions that measured gestational weight at last follow-up < 36 weeks on average compared to those that measured gestational weight at ≥36 on the outcome of weight.

### Data synthesis

Meta-analyses were conducted to examine the impact of physical activity interventions on the outcomes of interest. Five trial authors [15, 25, 27, 28, 30] were contacted to request missing information or data. Two trial authors were unable to provide the requested physical activity data when data was displayed in a published graph [15], or the published data was unclear [25]. The paper from Huang et al. [25], contained a data formatting error in the results. This was discussed between review authors (KJS, VEK) and agreed that the error did not affect the data and should therefore be included in the meta-analyses. One trial author was unable to provide participant numbers for individual group follow-up data but provided total participant follow-up [27]; therefore, the total participant follow-up number was divided equally between the two groups to ensure as much of the available evidence was included. Additionally, when a study had more than one intervention group, the number of participants in the comparator group was divided by the number of intervention groups.

Where trials reported data for light, moderate and vigorous physical activity separately, data were combined to create results for the primary outcome of total physical activity and the secondary outcome of MVPA through summation of the means and calculating the standard deviation. Furthermore, where trials provided minutes of daily MVPA data, this was converted to weekly MVPA values by multiplying the means and standard deviations by 7. Where trials did not report differences between the groups at baseline and follow-up, a standardised formula was used to calculate mean changes and standard deviations [33], this included a correlation coefficient of 0.6 for both intervention and control groups.

Nine meta-analyses were conducted (see Table S5 and S6 for further information) using RevMan 5.4.1 [34] (The Cochrane Collaboration). The primary outcome was calculated using SMD because trials used different physical activity outcomes (e.g., steps per day and total physical activity) with values interpreted using Cohen’s d effect sizes of small (0.2), moderate (0.5), or large (0.8) [35, 36]. Inverse variance meta-analysis using random effects models were used because of the variation in the types of interventions assessed in reports. *I* ^2^ statistic was calculated to determine the degree of heterogeneity in analyses whereby a result of 0-40% was considered as not important, 30-60% considered as moderate heterogeneity, 50-90% considered as substantial heterogeneity and 75-100% represented as considerable heterogeneity [37]. For last follow-up analysis, the longest available timeframe was used in analyses, regardless of the length of the intervention.

## Results

**Fig. 1:**
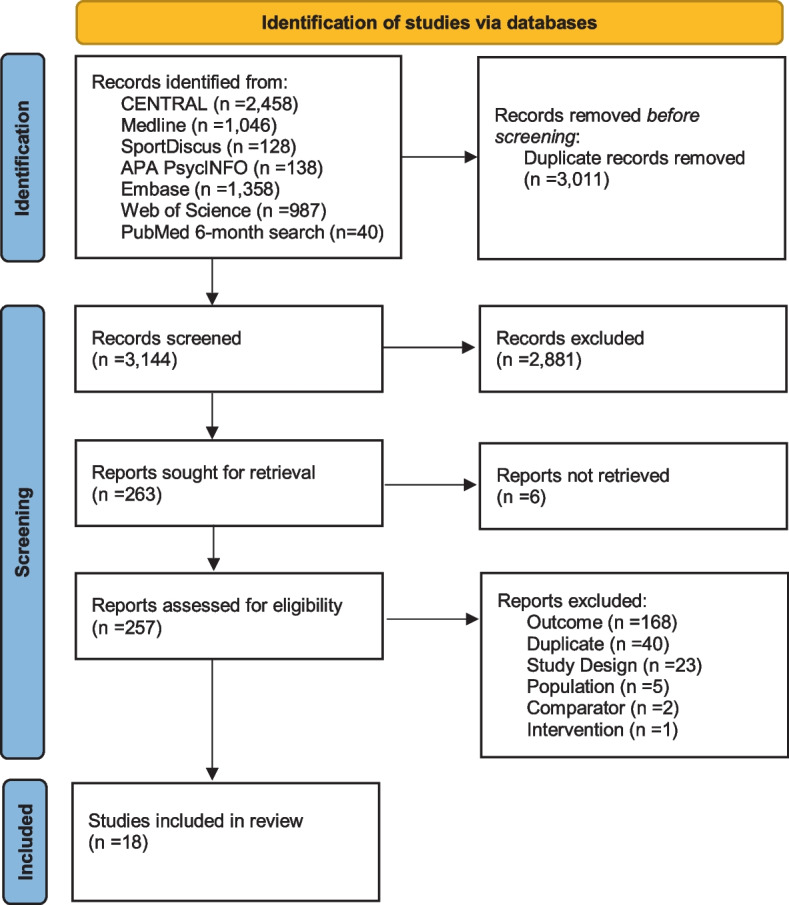
PRISMA (preferred reporting items for systematic review and meta-analyses flow diagram)

The searches identified 6155 potentially eligible publications, reducing to 3144 after duplicates were removed. Following title and abstract screening, 263 reports were screened as potentially eligible, and of these, six were inaccessible due to no author response, and 18 met the review eligibility criteria (Fig. 1). In these 18 studies, 1934 pregnant women were randomised to either a comparator (48.4%) or intervention (51.6%) group, with an overall retention rate of 76.5% with 754 and 725 women analysed in the intervention and comparator group, respectively.

### Characteristics of included trials

Most studies were conducted in the United States (*n* = 10) [17, 18, 20–22, 26, 27, 29–31] and Australia (*n* = 4) [15, 23–25] with the remainder in the UK (*n* = 2) [19, 28] and Canada (*n* = 2) [14, 16]. Trials recruited pregnant women with various body mass index (BMI) inclusion criteria. Studies restricted recruitment to women with a BMI ≥18.5 [17, 18, 29], or ≥ 19 [19], or ≥ 20 [25], or ≥ 25.0 [21, 22, 24, 26, 31], ≥30.0 [15, 16, 28], or ≤ 40.0 [14] with four trials not including a BMI restriction [20, 23, 27, 30]. For trials measuring gestational weight gain, seven [14–16, 21, 24, 25, 27] calculated this through directly measured weight by researchers; however, four [17, 22, 26, 29] calculated this based on participant measured pre-pregnancy weight and one did not specify the basis of the calculation [23]. Of the 18 included studies, those reporting ethnicity, education and marital status determined that 59% of the participants were White [16, 18, 20–23, 25, 28, 30, 31], with 75.1% graduating from higher education [14, 16, 18–22, 24, 25, 30, 31] and 88.9% living with partners [14, 16, 18–21, 28, 31].

Thirteen of the included interventions were categorised as multi-component lifestyle sessions that included physical activity [14, 15, 17, 19–22, 24, 25, 28–31]; however, five were exclusively physical activity interventions [16, 18, 23, 26, 27] aimed at promoting physical activity through a timetabled regime. Furthermore, intervention durations were primarily until birth [14, 15, 17, 19, 21, 22, 26, 27, 29–31] but 8- [28], 12- [16, 18, 20, 25] and 14-week [23, 24] interventions were also included. Furthermore, 66.7% of follow-up measurements took place at 34-38 weeks gestational age [14–17, 19, 25–31] with the remainder varying from intervention end [18, 23, 24] and other timepoints [20–22]; however, no last follow-up measurements were taken later than gestational week 38. Fourteen trials used accelerometers [14, 16–19, 21–23, 25, 26, 28–31] and four used pedometers [15, 20, 24, 27] to measure physical activity as a trial outcome. See Table S4 for details regarding the specific devices used.

### Synthesis of results

#### Total physical activity at last follow-up

**Fig. 2:**
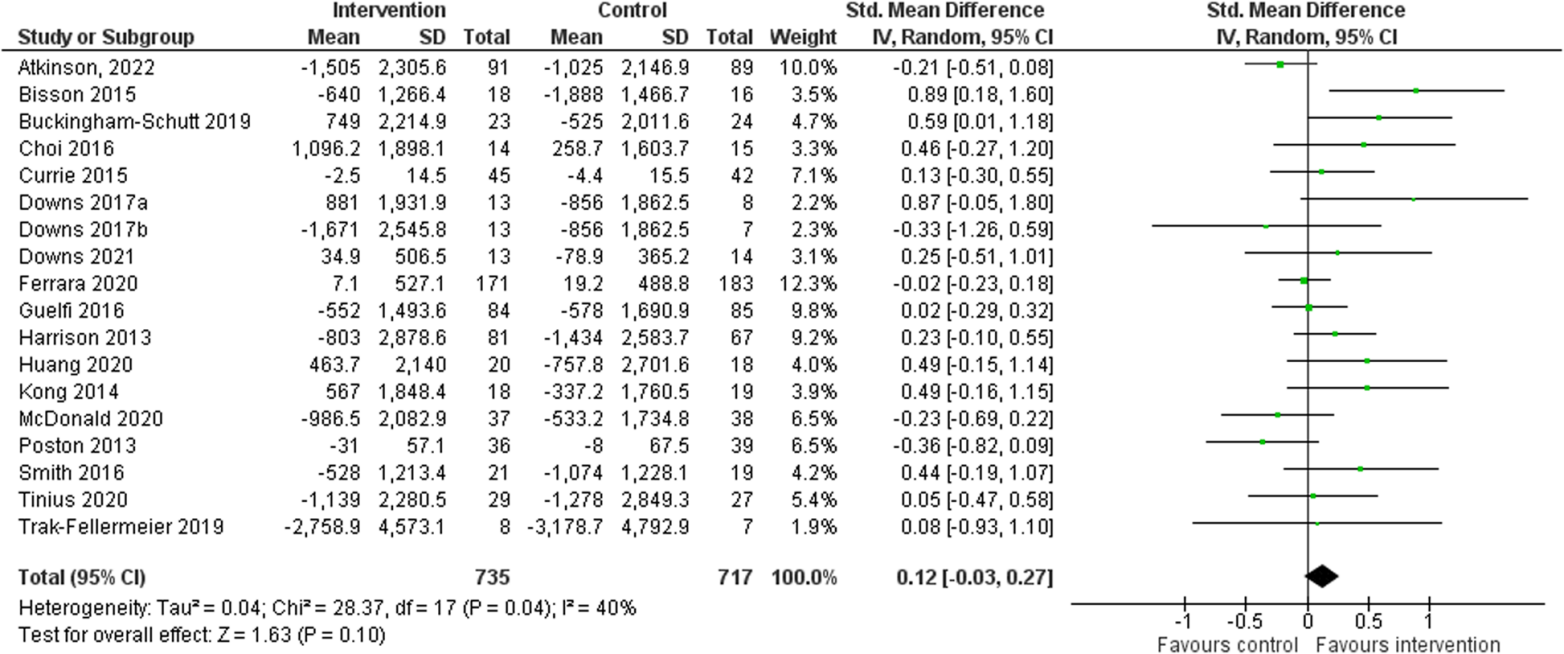
Standardised mean difference in total physical activity change at last follow-up

Seventeen trials measured the outcome of total physical activity [14, 16–31] via steps per day (*n* = 11) [14, 16–18, 20, 23–27, 30], minutes of light, moderate and vigorous physical activity per week (*n* = 2) [19, 28], vector magnitude (VM) counts per minute (n = 2) [22, 31], activity expenditure via kcal (*n* = 1) [21] and MET minutes per week (*n* = 1) [29]. No significant differences were found between the intervention and comparator group at last follow-up for total physical activity (95% confidence interval [CI] -0.03 to 0.27, *p* = 0.10, *I* ^2^ 40%) with a Cohen’s d effect of 0.12 [36]. See Fig. 2. Multi-component lifestyle interventions had a larger effect on total physical activity than exclusively physical activity-based interventions with Cohen’s d effect sizes of 0.16 (95% CI -0.02 to 0.34, *p* = 0.09, *I* ^2^ 37%) and 0.06 (95% CI -0.24 to 0.35, *p* = 0.71, *I* ^2^ 57%) respectively. See Fig. S1.

#### Total Physical activity at 24 to 30 weeks gestation (mid pregnancy)

Ten studies [14, 16–18, 24, 26–29, 31] measured total physical activity with data from 24 to 30 weeks gestation (see Fig. S2). No significant differences were found between the intervention and comparator group at this time point (95% CI -0.05 to 0.33, *p* = 0.15, *I* ^2^ 30%) with a Cohen’s d effect size of 0.14 [36].

#### Steps per day at last follow-up

Eleven trials [14, 16–18, 20, 23–27, 30] measured average total daily steps per day. At baseline, average steps per day were similar for the intervention and comparator groups (6704 and 6327 steps respectively). On average, women randomised to a physical activity intervention group achieved 435 (95% CI -0.5 to 870.6, *p* = 0.05, *I* ^2^ 54%) more steps per day than comparators at last follow-up (Fig. S3). These results represent 4.4 and 12.9% reductions in daily steps for the intervention and comparator groups at last follow-up, respectively.

#### Steps per day at 24 to 30 weeks gestation (mid pregnancy)

Seven trials [14, 16–18, 24, 26, 27] measured total steps per day at mid pregnancy. The intervention group, on average, achieved 7299 steps per day with comparators averaging 6162, equating to an average mean difference of 449 more steps per day favouring the intervention group (95% CI 5.5 to 892.7, *p* = 0.05, *I* ^2^ 35%) (see Fig. S4). These results convert into a 7.2% increase in daily steps and a 1% reduction for the intervention and comparator groups accordingly.

#### Moderate-to-vigorous intensity physical activity at last follow-up

Six trials [16, 19, 22, 26, 28, 29] were included in the meta-analysis for mean minutes of MVPA per week. No significant differences were found between the intervention and comparator group at last follow-up for MVPA (95% CI -4.3 to 44.2, *p* = 0.11) (See Fig. S5).

#### Moderate-to-vigorous intensity physical activity at 24 to 30 weeks gestation (mid pregnancy)

Four trials [16, 26, 28, 29] were included in the meta-analysis for mean minutes of MVPA per week. at mid pregnancy. At baseline, average MVPA minutes per week were similar for the intervention and comparator groups (140 and 129 minutes, respectively). On average, those randomised to a physical activity intervention group achieved 34.2 minutes (95% CI -0.5 to 68.9, *p* = 0.05, *I* ^2^ 53%) more MVPA per week than comparators at mid pregnancy (Fig. S6). These results represent a 12.8% increase for intervention and 16.3% reduction for the comparator group compared to baseline.

#### Gestational weight gain

**Fig. 3:**
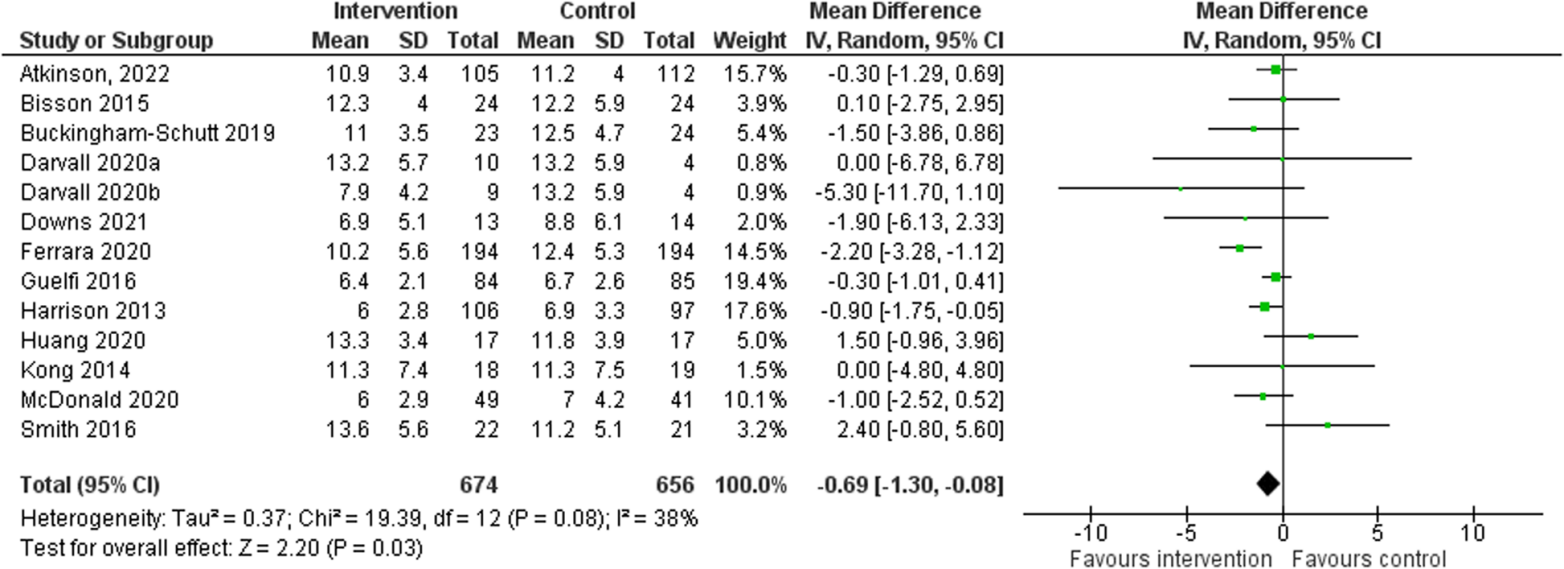
Mean difference in gestational weight gain (kg) change at last follow-up

A total of 12 [14–17, 21–27, 29] of the included trials also measured gestational weight gain (kg). The average baseline weight for women in the intervention and comparator groups were 80.2 kg and 80.3 kg, respectively. Women randomised to a physical activity intervention group on average gained 0.69 kg less weight than comparators at last follow-up (95% CI -1.30 to -0.08, *p* = 0.03, *I* ^2^ 38%) (See Fig. 3). Women randomised to a physical activity intervention group and weighed at < 36 weeks [17, 21, 23, 24, 26, 27, 29] gained 0.58 kg (95% CI -1.07 to 0.09, *p* = .02, *I* ^2^ 0%) less weight than comparators, and those weighed at ≥36 weeks [14–16, 22, 38] gained 0.78 kg (95% CI -2.12 to 0.73, *p* = 0.34, *I* ^2^ 61%) less weight than comparators. See Fig. S7.

### Risk of bias

All 18 trials were analysed for RoB. A total of 12 trials (66.7%) were at a high RoB, 4 (22.2%) of some concern, and 2 (11.1%) a low RoB (see Fig. S8). The main high RoB came from the measurement of the outcome (*n* = 9), with eight trials not reporting how long participants wore the physical activity measurement device for (e.g., valid wear time) and/or the threshold for the minimum amount of valid device wear to be included in the analyses. Additionally, when considering selection of the reported result, 14 studies were found to be of some concern because there was no previous publication of a specified analysis plan. For the five domains of the RoB2, the value for Cronbach’s Alpha was α = .66.

## Discussion

### Main findings

Previous research has reported that most pregnant women do not achieve sufficient amounts of physical activity during pregnancy, which may adversely affect their health [7]. Interventions that provide pregnant women with opportunities to participate in physical activity have been reported as beneficial to womens health but much of this evidence is based on self-reported physical activity data, which is prone to in accuracies and inflated effects due to recall bias and social desirability bias. For the first time, this systematic review and meta-analysis provides evidence that physical activity interventions during pregnancy have a small, but important, effect on increasing device-measured total steps per day and minutes of MVPA, relative to comparator groups. These interventions also reduced gestational weight gain by ~ 1 kg (95% CI -1.30 to -0.08). Guidance on this question can now be informed and updated by this evidence, which is based on more objective data than previous reviews.

### Physical activity (total, steps and MVPA)

Whilst many trials have been published, previous reviews have included trials that have assessed physical activity using both self-report and device measured physical activity data [7, 39, 40]. As such, it is difficult to make direct comparisons with other reviews, but our results are nonetheless similar to the systematic review of Currie et al. [40] who found that interventions focusing on physical activity behaviour reduced the decline in physical activity typically seen through pregnancy. The systematic review by Flannery et al. [39], focused on trials that had recruited pregnant women living with overweight and obesity and reported a larger SMD of 0.39 for physical activity (MET minutes per week) than seen in this review; although, 75% (*n* = 6) of their included studies included collected self-reported physical activity data. These results highlight the importance of drawing conclusions from using device-based measures of physical activity where the likely impact of physical activity interventions for pregnant women can be more accurately assessed. Of note, a systematic review of 15 RCTs, that aimed to investigate the efficacy of interventions to improve physical activity in pregnant women, concluded that many pregnant women do not achieve sufficient levels of physical activity because interventions promoting physical activity have only a small impact, similar to the modest findings of this review [7]. Nevertheless, it is important to put these results into a broader public health context and consider that an inverse dose-response relationship exists between MVPA and all-cause mortality, meaning that even small effects from physical activity interventions can still have an important effect on health, with further evidence of reduced risk of hypertensive disorders and total adverse maternal outcomes specific to this population [41–43]. Taken together, these studies highlight the importance of encouraging pregnant women to engage in physical activity throughout pregnancy and beyond childbirth.

There are several reasons why the effects of physical activity interventions might be relatively small in pregnant women. As pregnancy progresses, intrapersonal, interpersonal, and environmental barriers are perceived as increasingly difficult to overcome as fatigue, pregnancy discomfort, conflicting advice, and lack of access to specialist facilities often discourage women from being physically active [2]. These types of barriers become more salient and persuasive and dominates motivation to participate in the final months of pregnancy [2, 19]. With this in mind, it is expected that, even with intervention, women may complete less physical activity during the later stages of pregnancy. In a post-registration analysis, we explored the effects of physical activity interventions at 24 to 30 weeks gestation and found that the amount of total physical activity achieved by pregnant women at this stage of pregnancy was comparable to that found in the last follow up analysis. Previous research has reported that physical activity declines throughout pregnancy, although this conclusion is not necessarily supported by our results with similar effect sizes seen at 24 to 30 weeks (0.14) and last follow-up (0.12) for total physical activity; although women randomised to a physical activity intervention achieved significantly more minutes of MVPA per week than comparators (34 minutes) at 24-30 weeks of pregnancy, which reduced to 20 minutes at last follow-up and became a non-significant result. Additionally, women randomised to a physical activity intervention achieved significantly more steps per day with a 7.2% increase from baseline steps compared to a 1% reduction in steps for the comparator group at 24-30 weeks pregnancy; however, at last follow-up, both intervention and comparator groups reduced in steps per day by 4.4 and 12.9% compared to baseline, respectively. These results highlight the difficulties women may face in remaining physically active as pregnancy progresses. The final months of pregnancy are clearly a time where particular efforts need to be made to keep women physically active at a moderate intensity until they have given birth.

### Gestational weight gain

Pregnant women who had been randomised to a physical activity intervention weighed approximately ~ 1 kg (95% CI -1.30 to -0.08) less at the end of pregnancy than comparators. This finding is important because systematic reviews have reported that excessive gestational weight gain can increase the risk of gestational diabetes mellitus, with additional risks of caesarean birth and postnatal weight retention [44, 45]. Similar to this review, a meta-analysis of 12 RCTs, that aimed to determine whether physical activity could improve maternal and infant outcomes for overweight and obese pregnant women, reported that physical activity interventions, (using both self-report and device measures), reduced gestational weight gain by 1.14 kg (95% CI -1.67 to -0.62, *p* < 0.001, *I* ^2^ 10%) [38]. The discrepancy in weight gain (0.45 kg) difference between reviews may be related to the previous review recruiting only overweight and obese women. Nevertheless, findings from both reviews are important and relevant because with each 1 kg of gestational weight gained above the Institute of Medicine recommendations, pregnant women are 10% more likely to experience adverse pregnancy-related outcomes [24]. This finding supports the importance of promoting physical activity during pregnancy, both to promote physical activity, but also to manage excessive gestational weight gain and reduce the risk of adverse outcomes from pregnancy.

### Clinical Implications

Consistent with previous literature, our findings advocate in favour of the effects of physical activity interventions to promote physical activity during pregnancy, and to limit excessive gestational weight gain. Despite the significant increase in steps relative to comparators, many pregnant women continue to be unable to achieve the recommended guidelines of 150 minutes of moderate intensity physical activity per week, even after enrolment in a physical activity intervention. From a practical perspective, this review suggests that, on average, women randomised to a physical activity intervention group walked approximately 0.32 km more (435 steps) per day than comparators based on an average step length [46], which equates to an additional 2.24 km (~ 3045 steps) per week, and 9.92 km (~ 13,485 steps) for a 31-day month. Multi-component lifestyle interventions had a larger effect on total physical activity than exclusively physical activity interventions. Our findings reinforce the importance of physical activity during pregnancy within any lifestyle context, supporting the health message that all activity counts and is important for health [3]. Put into context, there were 613,936 live births in England and Wales in 2020 [47], and 3,613,647 live births in the United States in 2020 [48], meaning that participation in physical activity has the potential to influence the health of a substantial number of pregnant women, at a time where they may be more willing to engage in health behaviour change.

### Strengths and Limitations

This is the first systematic review and meta-analysis to focus exclusively on synthesising data from device-measured physical activity, rather than self-reported data, to report the health benefits of physical activity more accurately for pregnant women. Guidance can now be informed by this evidence that is based on more objective data. Comprehensive searches of published studies were conducted on six databases with no restrictions on language, date of publication or exclusion criteria of participant characteristics (e.g., BMI and age), enabling conclusions to be applied more broadly. With the primary focus of this review addressing total physical activity during pregnancy, the findings from this study contribute to the evidence supporting the most recent guidelines promoting the directive that all physical activity is important for health, regardless of intensity [3].

This review also has some limitations. We note that in some cases the significance value and corresponding 95% confidence intervals are exactly on the cusp of statistical significance and, therefore, need to be interpreted with some caution. The primary outcome of the review focused on total physical activity for two reasons. Physical activity guidelines in pregnant women indicate that all physical activity, regardless of intensity is beneficial for maternal and infant health and because vigorous intensity (e.g., running) physical activity is unlikely to be achieved and/or sustained during pregnancy [3]. Following on, trials were excluded if they only included data relating to MVPA and did not include data related to the primary outcome of total physical activity; therefore, findings related to MVPA are based only on a limited number of trials. This review specifically focused on device-measured physical activity, and this approach may not capture water-based physical activity as some devices (version of devices) are not waterproof. Swimming is a non-weight bearing activity favoured by pregnant women for health and is highly recommended to pregnant women by healthcare professionals; therefore, this limitation is particularly relevant here than might be the case with other populations [49, 50].

The RoB assessment suggests that our findings should be considered with some caution. Evidence from research with pregnant women acknowledges that ≥8 hours/day and ≥ 3 days/week of device wear time is needed to ensure higher accuracy and validity of device-measured data [19, 51]. As wear time is fundamental when reporting device-measured physical activity, eight (44.4%) trials providing no information or pre-specified cut-off for the minimum duration of device wear time and were thus adjudged as a high RoB. Consequently, as highlighted in the RoB assessment, it is imperative that future research with device-based measurements report their wear-time cut-off criteria and the specific model and placement of the device to allow for further subgroup analyses and study comparisons.

### Future Research Directions

In line with the primary outcome of this review, updated guidelines have highlighted the importance of promoting all durations and intensities of physical activity during pregnancy. With support from the findings of this review, future research should focus on promoting physical activity through simple objectives such as increasing steps per day, which can be achieved through shorter bouts of physical activity to overcome the barriers such as time, fatigue, and accessibility concerns associated with pregnancy.

With devices becoming more feasible, and thus more pervasive in the literature, researchers should prioritise RCTs with data collected from devices such as accelerometers to increase the reliability and validity of results for implementation. Advancements in wearable technology and analytical approaches should mean that in the future more studies are able to capture activities such as swimming more accurately via devices. Lastly, in future research involving pregnant populations, interventions measuring total physical activity should be prioritised over assessments of MVPA, consistent with the recommendation that every activity and minute of activity counts [3].

## Conclusion

When measured using a device, physical activity interventions delivered during pregnancy produce a small but important effect on increasing the number of steps per day relative to comparators and reduce the likelihood of excessive gestational weight gain. These findings are of health significance as previous research has demonstrated that physical activity declines through pregnancy. Both pregnant women and healthcare professionals can now be reassured of the benefits, as well as the likely magnitude of effect from participation in physical activity based on more objective data.

## Supplementary Information

**Additional file 1: Table S1.** PRISMA 2020 checklist. **Table S2.** PICO inclusion and exclusion criteria. **Table S3.** Medline (EBSCO), SportDiscus (EBSCO), APA PsycINFO (EBSCO), CENTRAL, Embase and Web of Science search terms. **Table S4.** Characteristics of included trials. **Table S5.** Explanation of meta-analyses for the primary outcome. **Table S6.** Explanation of meta-analyses for the secondary outcomes. **Fig. S1.** Standardised mean difference in total physical activity change at last follow-up with intervention type subgroups. **Fig. S2.** Standardised mean difference in total physical activity change at 24 to 30 weeks gestation. **Fig. S3.** Mean difference in steps per day change at last follow-up. **Fig. S4.** Mean difference in steps per day change at 24-30 weeks gestation. **Fig. S5.** Mean difference in MVPA change at last follow-up. **Fig. S6.** Mean difference in MVPA change at 24-30 weeks gestation. **Fig. S7.** Mean difference in gestational weight gain (kg) change at last follow-up with final measurement week subgroups. **Fig. S8.** Risk of bias assessments.

## Data Availability

All data generated during this study are included in this published article and its supplementary information file. The search strategy used in each database is shown in the supplementary information file with additional information regarding the individual databases searched and dates used within this published article.

## Abbreviations

BASE: Bielefeld Academic Search Engine
BMI: Body Mass Index
CENTRAL: Cochrane Central Register of Controlled Trials
CLiMB: Centre for Lifestyle Medicine and Behaviour
CI: Confidence Interval
MD: Mean Difference
MVPA: Moderate-Vigorous Physical Activity
NIHR: National Institute for Health and Care Research
PPAQ: Pregnancy Physical Activity Questionnaire
RCT(s): Randomised Controlled Trial(s)
RoB: Risk of Bias
SMD: Standardised Mean Difference
VM: Vector Magnitude
WHO: World Health Organisation

## References

[1] Bull FC, Al-Ansari SS, Biddle S, Borodulin K, Buman MP, Cardon G (2020). World Health Organization 2020 guidelines on physical activity and sedentary behaviour. Br J Sports Med.

[2] Harrison AL, Taylor NF, Shields N, Frawley HC (2018). Attitudes, barriers and enablers to physical activity in pregnant women: a systematic review. J Physiother.

[3] UK Chief Medical Officers’ Physical Activity Guidelines; 2019 [cited 2022 Mar 30]. Available from: https://assets.publishing.service.gov.uk/government/uploads/system/uploads/attachment_data/file/1054538/physical-activity-for-pregnant-women.pdf.

[4] Ferraro ZM, Gaudet L, Adamo KB (2012). The potential impact of physical activity during pregnancy on maternal and neonatal outcomes. Obstet Gynecol Surv.

[5] Ruifrok AE, Althuizen E, Oostdam N, van Mechelen W, Mol BW, de Groot CJM, et al. The relationship of objectively measured physical activity and sedentary behaviour with gestational weight gain and birth weight. J Pregnancy. 2014; Available from: https://pubmed.ncbi.nlm.nih.gov/25309754/. Cited 2022 Mar 30. 10.1155/2014/567379 PMC4189770 25309754

[6] de Oliveira CS, dos Imakawa TS, ECD M. Physical Activity during Pregnancy: Recommendations and Assessment Tools. Rev Bras Ginecol Obstet. 2017;39(8):424–32. 10.1055/s-0037-1604180 PMC10316950 28783859

[7] James P, Morgant R, Merviel P, Saraux A, Giroux-Metges MA, Guillodo Y, et al. How to promote physical activity during pregnancy: a systematic review. J Gynecol Obstet Hum Reprod. 2020;49(9):101864. 10.1016/j.jogoh.2020.101864 32663651

[8] McCarthy H, Potts HWW, Fisher A. Physical activity behavior before, during, and after COVID-19 restrictions: Longitudinal smartphone-tracking study of adults in the United Kingdom. J Med Internet Res. 2021;23(2):e23701. 10.2196/23701 PMC7861037 33347421

[9] World Health Organization (2018). Global action plan on physical activity 2018- 2030: more active people for a healthier world.

[10] Page MJ, McKenzie JE, Bossuyt PM, Boutron I, Hoffmann TC, Mulrow CD (2021). The PRISMA 2020 statement: an updated guideline for reporting systematic reviews. Syst Rev.

[11] Eustis EH, Ernst S, Sutton K, Battle CL (2019). Innovations in the Treatment of Perinatal Depression: the Role of Yoga and Physical Activity Interventions During Pregnancy and Postpartum. Curr Psychiatry Rep.

[12] Kettle VE, Madigan CD, Coombe A, Graham H, Thomas JJC, Chalkley AE, et al. Effectiveness of physical activity interventions delivered or prompted by health professionals in primary care settings: systematic review and meta-analysis of randomised controlled trials. BMJ. 2022:376:e068465. 10.1136/bmj-2021-068465 PMC8864760 35197242

[13] Covidence systematic review software, Veritas Health Innovation. Melbourne, Australia. Available from: www.covidence.org. Cited 2022 Mar 30

[14] Atkinson SA, Maran A, Dempsey K, Perreault M, Vanniyasingam T, Phillips SM (2022). Be Healthy in Pregnancy (BHIP): a randomized controlled trial of nutrition and exercise intervention from early pregnancy to achieve recommended gestational weight gain. Nutrients.

[15] Darvall JN, Wang A, Nazeem MN, Harrison CL, Clarke L, Mendoza C, et al. A pedometer-guided physical activity intervention for obese pregnant women (the Fit MUM Study): randomized feasibility study. JMIR Mhealth Uhealth. 2020;8(5):e15112. 10.2196/15112 PMC7284400 32348280

[16] Bisson M, Alméras N, Dufresne SS, Robitaille J, Rhéaume C, Bujold E, et al. A 12-week exercise program for pregnant women with obesity to improve physical activity levels: an open randomised preliminary study. PLoS One. 2015;10(9):e0137742. 10.1371/journal.pone.0137742 PMC4573757 26375471

[17] Buckingham-Schutt LM, Ellingson LD, Vazou S, Campbell CG (2019). The Behavioral Wellness in Pregnancy study: A randomized controlled trial of a multi-component intervention to promote appropriate weight gain. Am J Clin Nutr.

[18] Choi JW, Hyeon LJ, Vittinghoff E, Fukuoka Y (2016). mHealth Physical Activity Intervention: A Randomized Pilot Study in Physically Inactive Pregnant Women. Matern Child Health J.

[19] Currie S, Sinclair M, Liddle DS, Nevill A, Murphy MH (2015). Application of objective physical activity measurement in an antenatal physical activity consultation intervention: a randomised controlled trial. BMC Public Health.

[20] Downs D, DiNallo JM, Birch LL, Paul IM, Ulbrecht JS (2017). Randomized Face-to-face vs. home exercise interventions in pregnant women with gestational diabetes. Psychol Sport Exerc.

[21] Downs DS, Savage JS, Rivera DE, Pauley AM, Leonard KS, Hohman EE (2021). Adaptive, behavioral intervention impact on weight gain, physical activity, energy intake, and motivational determinants: results of a feasibility trial in pregnant women with overweight/obesity. J Behav Med.

[22] Ferrara A, Hedderson MM, Brown SD, Ehrlich SF, Tsai AL, Feng J (2020). A telehealth lifestyle intervention to reduce excess gestational weight gain in pregnant women with overweight or obesity (GLOW): a randomised, parallel-group, controlled trial. Lancet Diabetes Endocrinol.

[23] Guelfi KJ, Ong MJ, Crisp NA, Fournier PA, Wallman KE, Grove JR (2016). Regular Exercise to Prevent the Recurrence of Gestational Diabetes Mellitus: a Randomized Controlled Trial. Obstet Gynecol.

[24] Harrison CL, Lombard CB, Strauss BJ, Teede HJ (2013). Optimizing healthy gestational weight gain in women at high risk of gestational diabetes: A randomized controlled trial. Obesity.

[25] Huang RC, Silva D, Beilin L, Neppe C, Mackie KE, Roffey E (2020). Feasibility of conducting an early pregnancy diet and lifestyle e-health intervention: the Pregnancy Lifestyle Activity Nutrition (PLAN) project. J Dev Orig Health Dis.

[26] Kong KL, Campbell CG, Foster RC, Peterson AD, Lanningham-Foster L (2014). A pilot walking program promotes moderate-intensity physical activity during pregnancy. Med Sci Sports Exerc.

[27] McDonald SM, Yeo SA, Liu J, Wilcox S, Sui X, Pate RR (2020). Association between change in maternal physical activity during pregnancy and infant size, in a sample overweight or obese women. Women Health.

[28] Poston L, Briley AL, Barr S, Bell R, Croker H, Coxon K (2013). Developing a complex intervention for diet and activity behaviour change in obese pregnant women (the UPBEAT trial); Assessment of behavioural change and process evaluation in a pilot randomised controlled trial. BMC Pregnancy Childbirth.

[29] Smith K, Lanningham-Foster L, Welch A, Campbell C (2016). Web-based behavioral intervention increases maternal exercise but does not prevent excessive gestational weight gain in previously sedentary women. J Phys Act Health.

[30] Tinius R, Edens K, Link K, Susan Jones M, Lyons S, Rebelle T (2020). Effect of evidence-based materials and access to local resources on physical activity levels, beliefs, and motivation during pregnancy in a rural setting. J Phys Act Health.

[31] Trak-Fellermeier MA, Campos M, Meléndez M, Pomeroy J, Palacios C, Rivera-Viñas J (2019). Pearls randomized lifestyle trial in pregnant hispanic women with overweight/obesity: Gestational weight gain and offspring birthweight. Diabetes Metab Syndr Obes.

[32] Sterne JAC, Savović J, Page MJ, Elbers RG, Blencowe NS, Boutron I, et al. RoB 2: a revised tool for assessing risk of bias in randomised trials. BMJ. 2019;366 Available from: https://pubmed.ncbi.nlm.nih.gov/31462531/. Cited 2022 Mar 30. 10.1136/bmj.l4898 31462531

[33] Madigan CD, Fong M, Howick J, Kettle V, Rouse P, Hamilton L, et al. Effectiveness of interventions to maintain physical activity behavior (device-measured): Systematic review and meta-analysis of randomized controlled trials. Obes Rev. 2021;22(10):e13304. 10.1111/obr.13304 34129276

[34] The Cochrane Collaboration. Review Manager (RevMan) [Computer program]. Version 5.4. 2020.

[35] Andrade C (2020). Mean Difference, Standardized Mean Difference (SMD), and their use in meta-analysis: as simple as it gets. J Clin Psychiatry.

[36] Cohen J (2013). Statistical power analysis for the behavioral sciences.

[37] Cochrane Handbook for Systematic Reviews of Interventions | Cochrane Training. Available from: https://training.cochrane.org/handbook. Cited 2022 Mar 30

[38] Du MC, Ouyang YQ, Nie XF, Huang Y, Redding SR (2019). Effects of physical exercise during pregnancy on maternal and infant outcomes in overweight and obese pregnant women: a meta-analysis. Birth.

[39] Flannery C, Fredrix M, Olander EK, McAuliffe FM, Byrne M, Kearney PM (2019). Effectiveness of physical activity interventions for overweight and obesity during pregnancy: a systematic review of the content of behaviour change interventions. Int J Behav Nutr Phys Activity.

[40] Currie S, Sinclair M, Murphy MH, Madden E, Dunwoody L, Liddle D. Reducing the decline in physical activity during pregnancy: a systematic review of behaviour change interventions. PLoS One. 2013;8(6):e66385. 10.1371/journal.pone.0066385 PMC3682976 23799096

[41] Oja P, Kelly P, Murtagh EM, Murphy MH, Foster C, Titze S (2018). Effects of frequency, intensity, duration and volume of walking interventions on CVD risk factors: a systematic review and meta-regression analysis of randomised controlled trials among inactive healthy adults. Br J Sports Med.

[42] 2018 Physical Activity Guidelines Advisory Committee (2018). 2018 Physical Activity Guidelines Advisory Committee Scientific Report.

[43] Teede HJ, Bailey C, Moran LJ, Bahri Khomami M, Enticott J, Ranasinha S (2022). Association of antenatal diet and physical activity-based interventions with gestational weight gain and pregnancy outcomes: a systematic review and meta-analysis. JAMA Intern Med.

[44] McDowell M, Cain MA, Brumley J (2019). Excessive gestational weight gain. J Midwifery Womens Health.

[45] Kheirouri S, Alizadeh M (2020). Maternal excessive gestational weight gain as a risk factor for autism spectrum disorder in offspring: a systematic review. BMC Pregnancy Childbirth.

[46] Gottschall JS, Sheehan RC, Downs DS (2013). Pregnant women exaggerate cautious gait patterns during the transition between level and hill surfaces. J Electromyogr Kinesiol.

[47] Births in England and Wales - Office for National Statistics. Available from: https://www.ons.gov.uk/peoplepopulationandcommunity/birthsdeathsandmarriages/livebirths/bulletins/birthsummarytablesenglandandwales/2020. Cited 2022 Jun 29

[48] NVSS - Birth Data. Available from: https://www.cdc.gov/nchs/nvss/births.htm. Cited 2022 Jun 29

[49] Hayman M, Reaburn P, Alley S, Cannon S, Short C. What exercise advice are women receiving from their healthcare practitioners during pregnancy? Women Birth. 2020;33(4):e357–62. 10.1016/j.wombi.2019.07.302 31466828

[50] Parker JK, Angel L, Geoff P (2018). A qualitative examination of a mothers’ swim program: what keeps them coming back and how does it improve their psychological wellbeing?. Int J Womens Health Wellness.

[51] da Silva DF, Mohammad S, Nagpal TS, Scremin Souza SC, Colley RC, Adamo KB (2021). How many valid days are necessary to assess physical activity data from accelerometry during pregnancy?. J Phys Act Health.

