# Supplementary material for: Effectiveness of interventions to increase device-measured physical activity in pregnant women: systematic review and meta-analysis of randomised controlled trials
Source: Int J Behav Nutr Phys Act. 2022 Dec 1;19:142. doi: 10.1186/s12966-022-01379-w (PMC9713992; doi:10.1186/s12966-022-01379-w)
Supplement: Supplementary file 1 — Additional file 1: Table S1. PRISMA 2020 checklist. Table S2. PICO inclusion and exclusion criteria. Table S3. Medline (EBSCO), SportDiscus (EBSCO), APA PsycINFO (EBSCO), CENTRAL, Embase and Web of Science search terms. Table S4. Characteristics of included trials. Table S5. Explanation of meta-analyses for the primary outcome. Table S6. Explanation of meta-analyses for the secondary outcomes. Fig. S1. Standardised mean difference in total physical activity change at last follow-up with intervention type subgroups. Fig. S2. Standardised mean difference in total physical activity change at 24 to 30 weeks gestation. Fig. S3. Mean difference in steps per day change at last follow-up. Fig. S4. Mean difference in steps per day change at 24-30 weeks gestation. Fig. S5. Mean difference in MVPA change at last follow-up. Fig. S6. Mean difference in MVPA change at 24-30 weeks gestation. Fig. S7. Mean difference in gestational weight gain (kg) change at last follow-up with final measurement week subgroups. Fig. S8. Risk of bias assessments. [file 12966_2022_1379_MOESM1_ESM.docx]

**Supporting information**

**Effectiveness of interventions to increase device-measured physical activity in pregnant women: Systematic review and meta-analysis of randomised controlled trials**

^1^Kayleigh J Sharp, ^1^Lauren B Sherar, ^1^Victoria E Kettle, ^1^James P Sanders, ^1^Amanda J Daley

^1^Centre for Lifestyle Medicine and Behaviour (CLiMB), School of Sport Exercise and Health Sciences, Loughborough University, Leicestershire, LE11 3TU.

| **Table S1.** PRISMA 2020 checklist | | | |
| --- | --- | --- | --- |
| Section and Topic | Item # | Checklist item | Location where item is reported |
| **TITLE** | | | |
| Title | 1 | Identify the report as a systematic review. | Page 1 |
| **ABSTRACT** | | | |
| Abstract | 2 | See the PRISMA 2020 for Abstracts checklist. | Page 4 |
| **INTRODUCTION** | | | |
| Rationale | 3 | Describe the rationale for the review in the context of existing knowledge. | Page 6 |
| Objectives | 4 | Provide an explicit statement of the objective(s) or question(s) the review addresses. | Page 7 |
| **METHODS** | | | |
| Eligibility criteria | 5 | Specify the inclusion and exclusion criteria for the review and how studies were grouped for the syntheses. | Page 7 and 8 |
| Information sources | 6 | Specify all databases, registers, websites, organisations, reference lists and other sources searched or consulted to identify studies. Specify the date when each source was last searched or consulted. | Page 8 |
| Search strategy | 7 | Present the full search strategies for all databases, registers and websites, including any filters and limits used. | Table S3 |
| Selection process | 8 | Specify the methods used to decide whether a study met the inclusion criteria of the review, including how many reviewers screened each record and each report retrieved, whether they worked independently, and if applicable, details of automation tools used in the process. | Page 8 and 9 |
| Data collection process | 9 | Specify the methods used to collect data from reports, including how many reviewers collected data from each report, whether they worked independently, any processes for obtaining or confirming data from study investigators, and if applicable, details of automation tools used in the process. | Page 8 and 9 |
| Data items | 10a | List and define all outcomes for which data were sought. Specify whether all results that were compatible with each outcome domain in each study were sought (e.g., for all measures, time points, analyses), and if not, the methods used to decide which results to collect. | Page 9 and 10 |
|  | 10b | List and define all other variables for which data were sought (e.g., participant and intervention characteristics, funding sources). Describe any assumptions made about any missing or unclear information. | Page 9 and 10 |
| Study risk of bias assessment | 11 | Specify the methods used to assess risk of bias in the included studies, including details of the tool(s) used, how many reviewers assessed each study and whether they worked independently, and if applicable, details of automation tools used in the process. | Page 9 |
| Effect measures | 12 | Specify for each outcome the effect measure(s) (e.g., risk ratio, mean difference) used in the synthesis or presentation of results. | Page 11 |
| Synthesis methods | 13a | Describe the processes used to decide which studies were eligible for each synthesis (e.g., tabulating the study intervention characteristics and comparing against the planned groups for each synthesis (item #5)). | Page 11 |
|  | 13b | Describe any methods required to prepare the data for presentation or synthesis, such as handling of missing summary statistics, or data conversions. | Page 10 and 11 |
|  | 13c | Describe any methods used to tabulate or visually display results of individual studies and syntheses. | n/a |
|  | 13d | Describe any methods used to synthesize results and provide a rationale for the choice(s). If meta-analysis was performed, describe the model(s), method(s) to identify the presence and extent of statistical heterogeneity, and software package(s) used. | Page 11 |
|  | 13e | Describe any methods used to explore possible causes of heterogeneity among study results (e.g., subgroup analysis, meta-regression). | Page 11 |
|  | 13f | Describe any sensitivity analyses conducted to assess robustness of the synthesized results. | n/a |
| Reporting bias assessment | 14 | Describe any methods used to assess risk of bias due to missing results in a synthesis (arising from reporting biases). | Page 9 |
| Certainty assessment | 15 | Describe any methods used to assess certainty (or confidence) in the body of evidence for an outcome. | n/a |
| **RESULTS** | | | |
| Study selection | 16a | Describe the results of the search and selection process, from the number of records identified in the search to the number of studies included in the review, ideally using a flow diagram. | Figure 1 |
|  | 16b | Cite studies that might appear to meet the inclusion criteria, but which were excluded, and explain why they were excluded. | n/a |
| Study characteristics | 17 | Cite each included study and present its characteristics. | Table S4 |
| Risk of bias in studies | 18 | Present assessments of risk of bias for each included study. | Figure S8 |
| Results of individual studies | 19 | For all outcomes, present, for each study: (a) summary statistics for each group (where appropriate) and (b) an effect estimate and its precision (e.g. confidence/credible interval), ideally using structured tables or plots. | Page 13-15 |
| Results of syntheses | 20a | For each synthesis, briefly summarise the characteristics and risk of bias among contributing studies. | n/a |
|  | 20b | Present results of all statistical syntheses conducted. If meta-analysis was done, present for each the summary estimate and its precision (e.g. confidence/credible interval) and measures of statistical heterogeneity. If comparing groups, describe the direction of the effect. | Page 13-15 |
|  | 20c | Present results of all investigations of possible causes of heterogeneity among study results. | n/a |
|  | 20d | Present results of all sensitivity analyses conducted to assess the robustness of the synthesized results. | n/a |
| Reporting of biases | 21 | Present assessments of risk of bias due to missing results (arising from reporting biases) for each synthesis assessed. | Page 15 |
| Certainty of evidence | 22 | Present assessments of certainty (or confidence) in the body of evidence for each outcome assessed. | Page 13-15 |
| **DISCUSSION** | | | |
| Discussion | 23a | Provide a general interpretation of the results in the context of other evidence. | Page 16 |
|  | 23b | Discuss any limitations of the evidence included in the review. | Page 20 and 21 |
|  | 23c | Discuss any limitations of the review processes used. | Page 20 and 21 |
|  | 23d | Discuss implications of the results for practice, policy, and future research. | Page 19 and 21 |
| **OTHER INFORMATION** | | | |
| Registration and protocol | 24a | Provide registration information for the review, including register name and registration number, or state that the review was not registered. | Page 7 |
|  | 24b | Indicate where the review protocol can be accessed, or state that a protocol was not prepared. | Page 7 |
|  | 24c | Describe and explain any amendments to information provided at registration or in the protocol. | n/a |
| Support | 25 | Describe sources of financial or non-financial support for the review, and the role of the funders or sponsors in the review. | Page 2 |
| Competing interests | 26 | Declare any competing interests of review authors. | Page 2 |
| Availability of data, code, and other materials | 27 | Report which of the following are publicly available and where they can be found, template data collection forms; data extracted from included studies; data used for all analyses; analytic code; any other materials used in the review. | n/a |

| **Table S2.** PICO inclusion and exclusion criteria | | |
| --- | --- | --- |
|  | Inclusion | Exclusion |
| Population | Pregnant women aged ≥18 years old, including those with or without high-risk pregnancy (i.e., multiple births, existing health conditions, living with overweight and obesity).  No restriction on BMI. | Adult postnatal women (and any other population). |
| Intervention | Any physical activity intervention frequency, intensity, time, and type.  Any setting.  Group based or individual intervention. | Interventions excluding physical activity.  Physical activity intervention aimed entirely at body conditioning (e.g., yoga and tai-chi). |
| Comparator | Standard care provided by midwives and healthcare professionals. |  |
| Outcomes | Total physical activity measured by devices as a primary or secondary outcome with baseline and follow-up data provided. | Total physical activity measured via questionnaires.  Device assessed physical activity exclusively measuring moderate-to-vigorous physical activity (MVPA). |
| Studies | Randomised controlled trials and/or quasi-randomised controlled trials. | Studies of non-randomised controlled design (other study designs). |

| **Table S3.** Medline (EBSCO), SportDiscus (EBSCO), APA PsycINFO (EBSCO), CENTRAL, Embase and Web of Science search terms | |
| --- | --- |
| 1. | (Pregnan* OR pregnant wom?n OR antenatal OR prenatal OR prenatal period OR expectant mothers OR pregnant N3 wom?n OR during N3 pregnan* OR antenatal N3 wom?n OR prenatal wom?n). ti,ab. |
| 2. | (Physical activity OR exercise OR physical N2 activity OR physical activity N3 intervention* OR physical activity N3 program* OR exercise N3 intervention* OR exercise N3 program* OR light N5 activity OR moderate N5 activity OR vigorous N5 activity OR increas* N5 physical activity OR improv* N5 physical activity OR higher N5 physical activity OR chang* N5 physical activity OR modif* N5 physical activity OR increas* N5 exercise OR improv* N5 exercise OR higher N5 exercise OR chang* N5 exercise or modif* N5 exercise OR reduc* N5 sedentary behav* OR decreas* N5 sedentary behav* OR lower* N5 sedentary behav* OR chang* N5 sedentary behav* OR modif* N5 sedentary behav*). ti,ab. |
| 3. | (Random* N3 assigned OR randomi?ed OR RCT OR quasi N2 RCT OR quasi N2 randomi?ed OR randomi?ed controlled trial). ti,ab. |
| 4. | 1 AND 2 AND 3 |

| **Table S4.** Characteristics of included trials | | | | |  | | |
| --- | --- | --- | --- | --- | --- | --- | --- |
| **Study ID**  **Country**  **Design** | **Participants**  Number, Age, BMI | **Intervention Component**/s | **Comparator**/s | **Baseline**  **Follow-up** | | **Outcomes Included** | **Measurement** |
| Atkinson, 2022 (14)  Canada  RCT | N= 241, 31.4 (4.1) yrs, 25.5 (4.6) kg/m^2^ | In-person counselling bi-weekly with nutritionist & walking-based program 3-4/week for 25mins & a goal of 10,000 steps/day. | Standard antenatal care. | B: 12-17 wks GA  F-up: 26-28 wks GA† & 36-38 wks GA* | | - Steps/day - GWG | - Accelerometer   (SenseWear armband MF-SW) |
| Bisson, 2015 (16)  Canada  RCT | N= 50, 30.8 (3.9) yrs, 34.3 (5.0) kg/m^2^ | 3 wkly 1-hr sessions in a hospital-based conditioning centre over 12-wks. 5-10 mins warm-up on stationary ergo cycle, 15-30 mins treadmill walk progressively increasing, 20 min muscular work out, & cool-down. | Standard antenatal care. | B: 14 wks GA  F-up: 28-wks GA† & 36-wks GA* | | - Steps/day - MVPA - GWG | - Accelerometer   (ActiGraph GT3X) |
| Buckingham-Schutt, 2019 (17)  USA  RCT | N= 47, 31.4 (4.1) yrs, 25.0 (4.5) kg/m^2^ | Monthly 15- to 30-min one-to-one visits & wkly contact with dietitian & walking goal of 10,000 steps/day on average until delivery. | Standard antenatal care. | B: 8-14 wks GA  F-up: 26-28 wks GA† & 34-36 wks GA* | | - Steps/day - GWG | - Accelerometer (SenseWear armband) |
| Choi, 2016 (18)  USA  RCT | N= 30, 33.7 (2.6) yrs, 27.7 (3.7) kg/m^2^ | Increase steps until ≥8500 steps/day ≥5 days p/w over 12-wks & daily text message or short video & daily activity diary. | Standard antenatal care & PA goals. | B: 10-20 wks GA  F-up: 5-8 wks after baseline† & 9-12 wks after baseline* | | - Steps/day | - Accelerometer   (Fitbit Ultra) |
| Currie, 2015 (19)  UK  RCT | N= 97, 31.1 (4.5) yrs, 25.8 (4.1) kg/m^2^ | 3 x 30-60 min individually tailored PA consultations, delivered by a researcher, encouraging 30 mins of moderate-intensity PA ≥5 days p/w until delivery. | Standard antenatal care. | B: 12-15 wks GA  F-up: 35-37 wks GA* | | - LVPA - MVPA | - Accelerometer   (ActiGraph- GT3X) |
| Darvall, 2020 (15)  Australia  RCT | N= 30, 29.5 (5.4) yrs, 36.5 (4.3) kg/m^2^ | Group 1:  App-coach: Initial 1-hr f2f behaviour change session with a health coach, then 3 follow-up 20-min phone calls at 24, 28 & 32 wks GA to review & reinforce strategies to increase steps until delivery.  Group 2:  App-only: Self-monitor steps/day & activity minutes via pedometer display & general PA guidelines until delivery. | Standard antenatal care & PA goals | B: 12-16 wks GA  F-up: 36-37 wks GA* | | - Steps/day - GWG | - Pedometer   (Fitbit Zip) |
| Downs, 2017 (20)  USA  RCT | N= 41, 32.0 (4.3) yrs, 29.1 (8.3) kg/m^2^ | Group 1:  F2F: 2 days p/w delivered 70-min walking/ low-impact aerobics session led by fitness instructor for 12-wks.  Group 2:  Home: Encouraged to exercise individually to meet guidelines & 45-min call every 2-wks from a certified fitness instructor. | Standard antenatal care. | B: 20 wks GA  F-up: 32 wks GA* | | - Steps/day | - Pedometer   (Piezoelectric NL1000) |
| Downs, 2021 (21)  USA  RCT | N= 31, 29.6 (4.1) yrs, 32.6 (7.2) kg/m^2^ | 24 x 45-60 min individually tailored sessions with dietician encouraging PA & 5 step program incorporating PA guidelines incl. walking/ low-impact aerobics sessions with fitness instructor & step goals until delivery. | Standard antenatal care. | B: 8-12 wks GA  F-up: 33-38 wks GA* | | - Activity expenditure kcal - GWG | - Accelerometer   (Jawbone UP3) |
| Ferrara, 2020 (22)  USA  RCT | N= 394, 32.5 (4.0) yrs, 29.4 (3.6) kg/m^2^ | 13 wkly, dietitian led, individual lifestyle sessions incorporating PA guidelines into weekly routine (150 min p/w MVPA). First & last session delivered F2F, remaining 11 delivered by phone. Optional maintenance sessions bi-wkly until delivery. | Standard antenatal care. | B: 8-15 wks GA  F-up: 29-38 wks GA* | | - Total VMCPM - MVPA - GWG | - Accelerometer   (ActiGraph WGT3x-BT) |
| Guelfi, 2016 (23)  Australia  RCT | N= 172, 33.7 (4.0) yrs, 26.0 (5.2) kg/m^2^ | 3 times p/w stationary cycling program supervised by exercise physiologist. 5-min warm-up, interval cycling, 5-min cool-down. Duration progressively increased by 5- min increments every 2-3 wks, from 20-30 mins to ≤60 mins. | Standard antenatal care. | B: 12-14 wks GA  F-up: 14 wks after baseline* | | - Steps/day - GWG | - Accelerometer (ActiGraph) |
| Harrison, 2013 (24)  Australia  RCT | N= 228, 32.1 (4.6) yrs, 30.4 (5.7) kg/m^2^ | 4 health coached delivered behaviour change lifestyle sessions (14-16, 20-, 24- & 28 wks GA) with individual PA goals reinforced by on-going support & contact through SMS. | Standard antenatal care & 1 education session. | B: 12-15 wks GA  F-up: 28 wks GA* | | - Steps/day - GWG | - Pedometer (Yamax Digiwalker SW-700) |
| Huang, 2020 (25)  Australia  RCT | N= 57, 33.3 (4.1) yrs, 25.7 (5.4) kg/m^2^ | 12-wks web-based program providing diet & PA advice. Wkly SMS contact, 1 private session with a dietitian regarding accelerometer data feedback & wkly modules released online. | Standard antenatal care. | B: 6-11 wks GA  F-up: 36-wks GA* | | - Steps/day - GWG | - Accelerometer   (ActiGraph WGT3x-BT) |
| Kong, 2014 (26)  RCT  USA | N= 37, 27.0 (3.9) yrs, 30.6 (2.7) kg/m^2^ | Unsupervised walking program from 12-15 wks GA until delivery. To accumulate 150 mins of moderate PA p/w with minimum bouts of ≥10-mins. | Standard antenatal care. | B: 10-14 wks GA  F-up: 27-29 wks GA† & 34-36 wks GA* | | - Steps/day - MVPA - GWG | - Accelerometer (StepWatch activity monitor) |
| McDonald,  2020 (27)  USA  RCT | N= 90, 32.3 (4.7) yrs, 30.0 (7.1) kg/m^2^ | Walking program of 40 mins, 5 times p/w at moderate intensity (55-69% of heart rate reserve) until delivery. | 40 min stretching program 5 times p/w. | B: 14-17 wks GA  F-up: 26-29 wks GA† & 34-37 wks GA* | | - Steps/day - GWG | - Pedometer   (Yamax Digiwalker SW200) |
| Poston, 2013 (28)  UK  RCT | N= 183, 30.5 (5.3) yrs, 36.3 (4.7) kg/m^2^ | One-to-one appt with health trainer & wkly group sessions for 8 wks. Encouraged to increase step count & create PA goals. | Standard antenatal care. | B: 15-17 wks GA  F-up: 36-37 wks GA* | | - LVPA - MVPA | - Accelerometer   (ActiGraph GT1M or GT3X) |
| Smith, 2016 (29)  USA  RCT | N= 51, 29.6 (4.5) yrs, 26.4 (4.6) kg/m^2^ | Web-based intervention with access to lifestyle modules, journal, calendar to track exercise, & community forum. Gradually work up to 150 minutes of moderate PA p/w (in ≥10-minute bouts) & sustain until delivery. | Standard antenatal care. | B: 10-14 wks GA  F-up: 24-26 wks GA† & 34-36 wks GA* | | - MET minutes - MVPA - GWG | - Accelerometer (SenseWear Mini armband MF-SW) |
| Tinius, 2020 (30)  USA  RCT | N= 70, 29.7 (4.9) yrs, 26.1 (6.2) kg/m^2^ | Access to evidence-based educational materials with local PA facilities provided free of charge until delivery. | Standard antenatal care. | B: 8-16 wks GA  F-up: 34-37 wks GA* | | - Steps/day | - Accelerometer   (Xiaomi Mi Band) |
| Trak-Fellermeier, 2019 (31)  USA  RCT | N= 31, 27.7 (5.5.) yrs, 35.3 (7.4) kg/m^2^ | Diet & PA intervention, delivered by dietician, to increase movement & reduce sedentary time until delivery with encouragement to set daily PA goals. | Standard antenatal care. | B: 8-16 wks GA  F-up: 35-36 wks GA* | | - Total VMCPM | - Accelerometer   (ActiGraph GT3X+) |
| BMI, body mass index; RCT, randomised controlled trial; N, number; yrs, years; wkly, weekly; wks, weeks; min/s, minute/s; B, baseline; F-up, follow-up; GA, gestational age; steps/day, steps per day; GWG, gestational weight gain; MVPA, moderate & vigorous physical activity; UK, United Kingdom; QRCT, quasi-randomised controlled trial; p/w, per week; USA, United States of America; PA, physical activity; LVPA, light to vigorous physical activity; F2F, face-to-face; incl., including; VMCPM, vector magnitude counts per minute; SMS, short message service; appt, appointment. †Follow-up value used in 24 to 30 weeks gestation meta-analyses; *Follow-up value used in last available follow-up meta-analyses. | | | | | | | |

| **Table S5.** Explanation of meta-analyses for the primary outcome | | | | | |
| --- | --- | --- | --- | --- | --- |
| Population | Follow-up | Outcome | Comparator | Completed:  Yes/no | Explanation |
| 1. Pregnant adult women | Last available follow-up | Total PA | Standard antenatal care | Yes |  |
| 1. Pregnant adult women | 24 to 30 weeks gestation | Total PA | Standard antenatal care | Yes |  |
| 1. Pregnant adult women | Last follow-up | Total PA with intervention type subgroups | Stanndard antenatal care | Yes |  |
| 1. Pregnant adult women | Last available follow-up | Steps/day | Standard antenatal care | Yes |  |
| 1. Pregnant adult women | 24 to 30 weeks gestation | Steps/day | Standard antenatal care | Yes |  |
| 1. Pregnant adult women | Last available follow-up | LVPA mins | Standard antenatal care | No | LVPA was measured differently in the two trials. |
| 1. Pregnant adult women | Last available follow-up | Total VMCPM | Standard antenatal care | No | Participant number varied drastically in the two trials (n=354, n=15). |
| 1. Pregnant adult women | Last available follow-up | MET mins | Standard antenatal care | No | Only one trial measured change in MET mins; therefore, meta-analysis was not possible. |
| 1. Pregnant adult women | Last available follow-up | Activity expenditure kcal | Standard antenatal care | No | Only one trial measured change in activity expenditure kcal; therefore, meta-analysis was not possible. |
| LVPA, light to vigorous physical activity; PA, physical activity; LPA, light physical activity; CPM, counts per minute; MPA, moderate physical activity; VPA, vigorous physical activity; total VMCPM, vector magnitude counts per minute; min/s, minute/s; MET, metabolic equivalent; kcal, kilocalories. | | | | | |

| **Table S6.** Explanation of meta-analyses for the secondary outcomes | | | | | | | |
| --- | --- | --- | --- | --- | --- | --- | --- |
| Population | Follow-up | Outcome | Comparator | Completed:  Yes/no | | Explanation | |
| 1. Pregnant adult women | Last available follow-up | MVPA mins/wk | Standard antenatal care | Yes |  | | |
| 1. Pregnant adult women | 24 to 30 weeks gestation | MVPA mins/wk | Standard antenatal care | Yes |  | |  |
| 1. Pregnant adult women | Last available follow-up | GWG (kg) | Standard antenatal care | Yes |  | | |
| 1. Pregnant adult women | Last available follow-up | GWG (kg) with final measurement week subgroups | Standard antenatal care | Yes |  | | |
| MVPA mins/wk, moderate-to-vigorous physical activity minutes per week; GWG, gestational weight gain. | | | | | | | |

**Figure S1:** Standardised mean difference in total physical activity change at last follow-up with intervention type subgroups


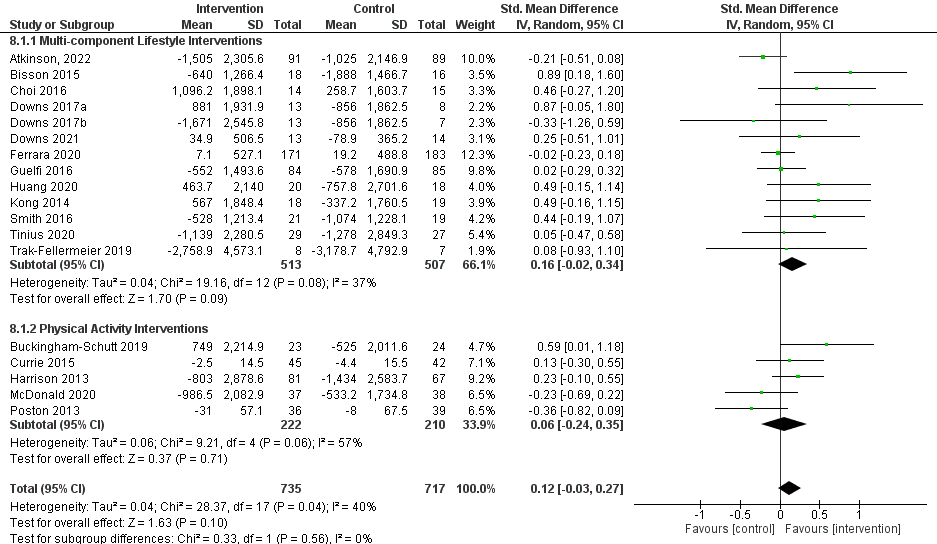


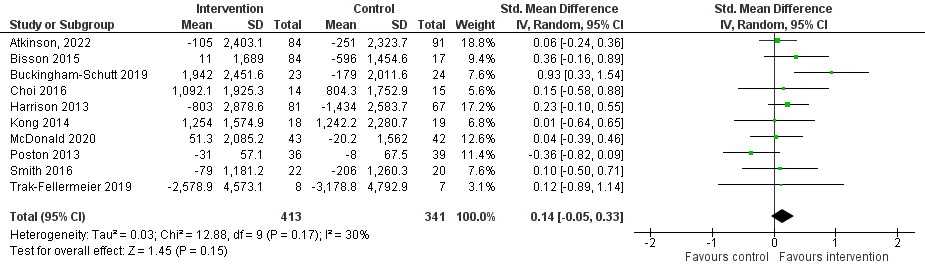
**Figure S2:** Standardised mean difference in total physical activity change at 24 to 30 weeks gestation

**Figure S3:** Mean difference in steps per day change at last follow-up

**
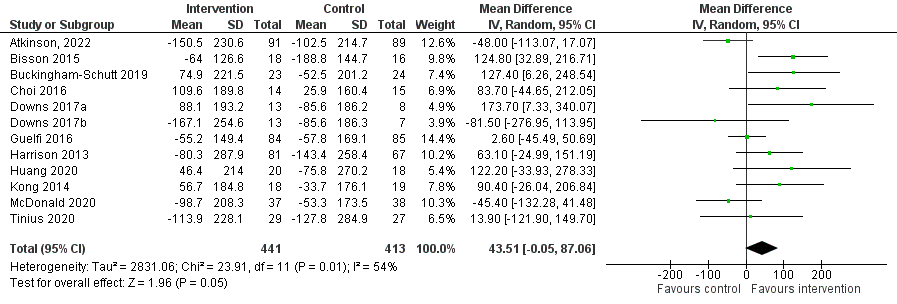
**

**For graphical purposes the numbers were divided by ten.*

**
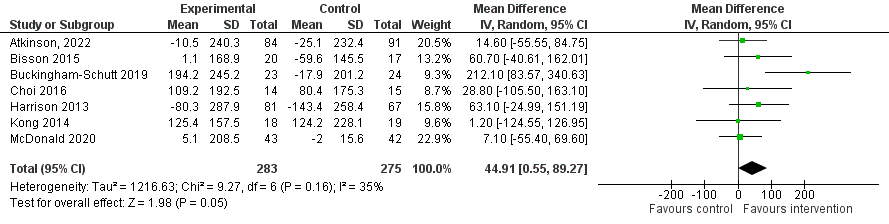
Figure S4:** Mean difference in steps per day change at 24-30 weeks gestation

**For graphical purposes the numbers were divided by ten.*

**Figure S5:** Mean difference in MVPA change at last follow-up


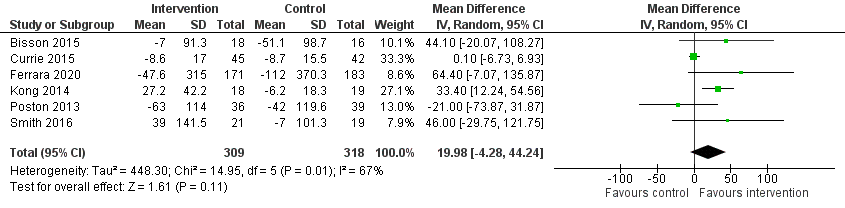


**Figure S6:** Mean difference in MVPA change at 24-30 weeks gestation


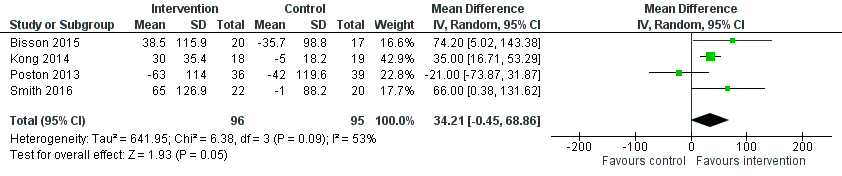


**Figure S7:** Mean difference in gestational weight gain (kg) change at last follow-up with final measurement week subgroups

**
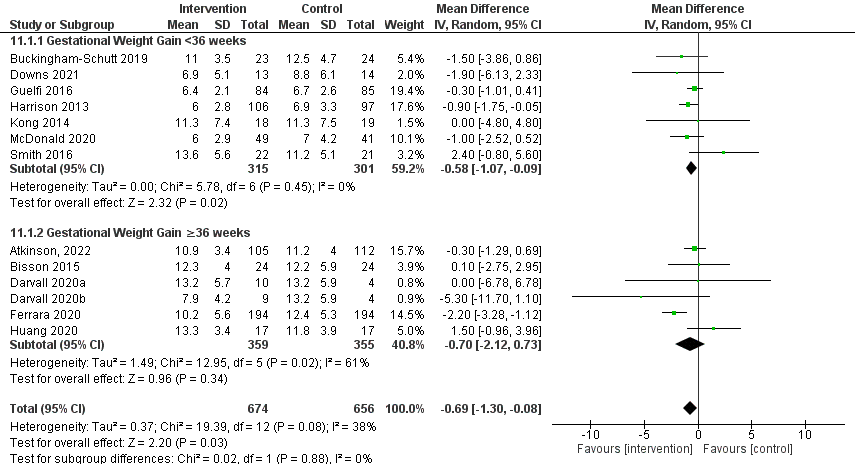
**

**Figure S8:** Risk of bias assessments

|  | **D1** | **D2** | **D3** | **D4** | **D5** | **Overall** |
| --- | --- | --- | --- | --- | --- | --- |
| Atkinson, 2022 |  |  |  |  |  |  |
| Bisson, 2015 |  |  |  |  |  |  |
| Buckingham-Schutt, 2019 |  |  |  |  |  |  |
| Choi, 2016 |  |  |  |  |  |  |
| Currie, 2015 |  |  |  |  |  |  |
| Darvall, 2020 |  |  |  |  |  |  |
| Downs, 2017 |  |  |  |  |  |  |
| Downs, 2021 |  |  |  |  |  |  |
| Ferrara, 2020 |  |  |  |  |  |  |
| Guelfi, 2016 |  |  |  |  |  |  |
| Harrison, 2013 |  |  |  |  |  |  |
| Huang, 2020 |  |  |  |  |  |  |
| Kong, 2014 |  |  |  |  |  |  |
| McDonald, 2020 |  |  |  |  |  |  |
| Poston, 2013 |  |  |  |  |  |  |
| Smith, 2016 |  |  |  |  |  |  |
| Tinius, 2020 |  |  |  |  |  |  |
| Trak-Fellermeier, 2019 |  |  |  |  |  |  |

Low risk

Some concerns

High risk

D1 Randomisation process

D2 Deviations from the intended interventions

D3 Missing outcome data

D4 Measurement of the outcome

D5 Selection of the reported result
